# Construction of a high-density genetic map and QTL localization of body weight and wool production related traits in Alpine Merino sheep based on WGR

**DOI:** 10.1186/s12864-024-10535-4

**Published:** 2024-06-27

**Authors:** Wentao Zhang, Zengkui Lu, Tingting Guo, Chao Yuan, Jianbin Liu

**Affiliations:** 1 Key Laboratory of Animal Genetics and Breeding on Tibetan Plateau, Ministry of Agriculture and Rural Affairs, Lanzhou Institute of Husbandry and Pharmaceutical Sciences, Chinese Academy of Agricultural Sciences, Lanzhou, 730050 China; 2 Sheep Breeding Engineering Technology Research Center of Chinese Academy of Agricultural Sciences, Lanzhou, 730050 China; 3 State Key Laboratory of Animal Biotech Breeding, Institute of Animal Sciences, Chinese Academy of Agricultural Sciences, Beijing, 100193 China

**Keywords:** Wool and weight traits, Genetic linkage mapping, QTL localization, Genome, SNP

## Abstract

**Background:**

The Alpine Merino is a new breed of fine-wool sheep adapted to the cold and arid climate of the plateau in the world. It has been popularized in Northwest China due to its superior adaptability as well as excellent production performance. Those traits related to body weight, wool yield, and wool fiber characteristics, which are economically essential traits in Alpine Merino sheep, are controlled by QTL (Quantitative Trait Loci). Therefore, the identification of QTL and genetic markers for these key economic traits is a critical step in establishing a MAS (Marker-Assisted Selection) breeding program.

**Results:**

In this study, we constructed the high-density genetic linkage map of Alpine Merino sheep by sequencing 110 F_1_ generation individuals using WGR (Whole Genome Resequencing) technology. 14,942 SNPs (Single Nucleotide Polymorphism) were identified and genotyped. The map spanned 2,697.86 cM, with an average genetic marker interval of 1.44 cM. A total of 1,871 high-quality SNP markers were distributed across 27 linkage groups, with an average of 69 markers per LG (Linkage Group). Among them, the smallest genetic distance is 19.62 cM for LG2, while the largest is 237.19 cM for LG19. The average genetic distance between markers in LGs ranged from 0.24 cM (LG2) to 3.57 cM (LG17). The marker density in the LGs ranged from LG14 (39 markers) to LG1 (150 markers).

**Conclusions:**

The first genetic map of Alpine Merino sheep we constructed included 14,942 SNPs, while 46 QTLs associated with body weight, wool yield and wool fiber traits were identified, laying the foundation for genetic studies and molecular marker-assisted breeding. Notably, there were QTL intervals for overlapping traits on LG4 and LG8, providing potential opportunities for multi-trait co-breeding and further theoretical support for selection and breeding of ultra-fine and meaty Alpine Merino sheep.

**Supplementary Information:**

The online version contains supplementary material available at 10.1186/s12864-024-10535-4.

## Background

Since long ago, China, as the world’s largest wool processing and consuming country [1–3], has relied up to 80% on imported wool, especially high-quality fine wool [4, 5]. Meanwhile, the large-scale shortage of high-quality fine wool has made China’s high-grade wool textile industry extremely dependent on the international wool market [2, 4, 6–9]. In the past twenty years, a substantial revolution in the quality of the world’s wool has occurred, mainly in the wool fineness from “coarse” to “fine”; the finer, the more valuable [10–12]. For now, China has been maintaining about 30 million fine-wool sheep with wool fiber diameter of 21.6–25.0 μm [13–15], but because past improvements were made to increase wool production (clean wool per sheep is only 1/4 of that of Australia, less than 1 kg, in 1986 [14]) rather than wool fiber diameter [16, 17]. Consequently, there are only a tiny number of fine-wool sheep with wool diameter less than 21.5 μm [5], which does not meet the demand of the wool spinning industry for high-grade fine wool of 21.5 μm [18]. The Alpine Merino sheep, created through advanced breeding techniques with Australian Merino as the sire and GanSu Alpine Merino [8, 19] as the dam, surpassed the wool quality level of the same type of Australian Merino. The successful breeding of Alpine Merino sheep has fulfilled the blank of fine-type fine-wool sheep breeding in high-altitude ecological zones in the world, realized the localization of Australian Merino sheep in high-altitude ecological zones in China, and enriched the genetic resource structure of Chinese sheep breeds. The breed is adapted to the ecological zone of 2400–4070 m, with good resistance and ecological differentiation advantages; the wool fineness reaches 19.1–21.5 μm; it retains the mother parent’s good adaptation to the ecological environment of high-altitude cold and arid mountainous areas, and inherits the characteristics of the father parent’s wool with high comprehensive quality.

As a new breed of fine-wool sheep, the body weight and wool production-related traits of Alpine Merino sheep are essential and complex quantitative traits with polygenic inheritance [20–24]. With the continuous improvement and cost reduction of NGS (Next Generation Sequencing) technologies [25], QTL analysis using SNPs from WGR has become an emerging approach [26, 27]. WGR SNP data offers greater marker densities, comprehensive genome coverage [27], robust detection of rare variants [28–31], higher resolution, and more extensive information than traditional QTL methods, revealing the genetic basis of numerous quantitative traits. QTL analysis based on WGR has been widely used in aquatic biology [32], such as *Takifugu rubripes* [33], *Andrias davidianus* [34], *Seriola dumerili* [35], *Oreochromis* [36] and *Oncorhynchus mykiss* [37]. Body weight and wool production related traits in sheep are economically important. A large number of studies (GWAS (Genome-Wide Association Study) based on high-density SNP chip and WGR technology) have reported genetic variation for these traits [22, 38, 39]. QTL analysis not only identifies tightly interlinked molecular markers, but also determines the location of genes involved in the regulation of complex quantitative traits [40, 41]. This provides a valuable resource for breeding and functional genomics research. However, relatively few QTL studies have been reported to date [23, 24, 42], and more comprehensive and detailed QTL studies based on high-throughput SNPs are especially rare. Several studies have established independent linkage maps to localize QTL [43–46], but most of these studies have been limited to partial genome scans. The construction of a whole genetic linkage map for Alpine Merino sheep is imperative to advance QTL analysis and understanding the genetic basis.

This study used seven parents and their 110 F_1_ generation individuals in Alpine Merino sheep as a mapping population. Genotyping was performed using WGR technology. The pedigree data were then used to construct a high-density genetic linkage map and to pinpoint important economic QTL traits, including three traits related to body weight, three traits related to wool yield, and six traits related to wool fiber characteristics. This study enables researchers to understand the genetic basis of Alpine Merino sheep in greater depth. The data obtained in this study will provide a powerful justification for applying molecular MAS in Alpine Merino sheep. It also provides valuable information for improving and breeding ultra-fine and meat strains of Alpine Merino sheep.

## Results

### Phenotype traits

**Fig. 1:**
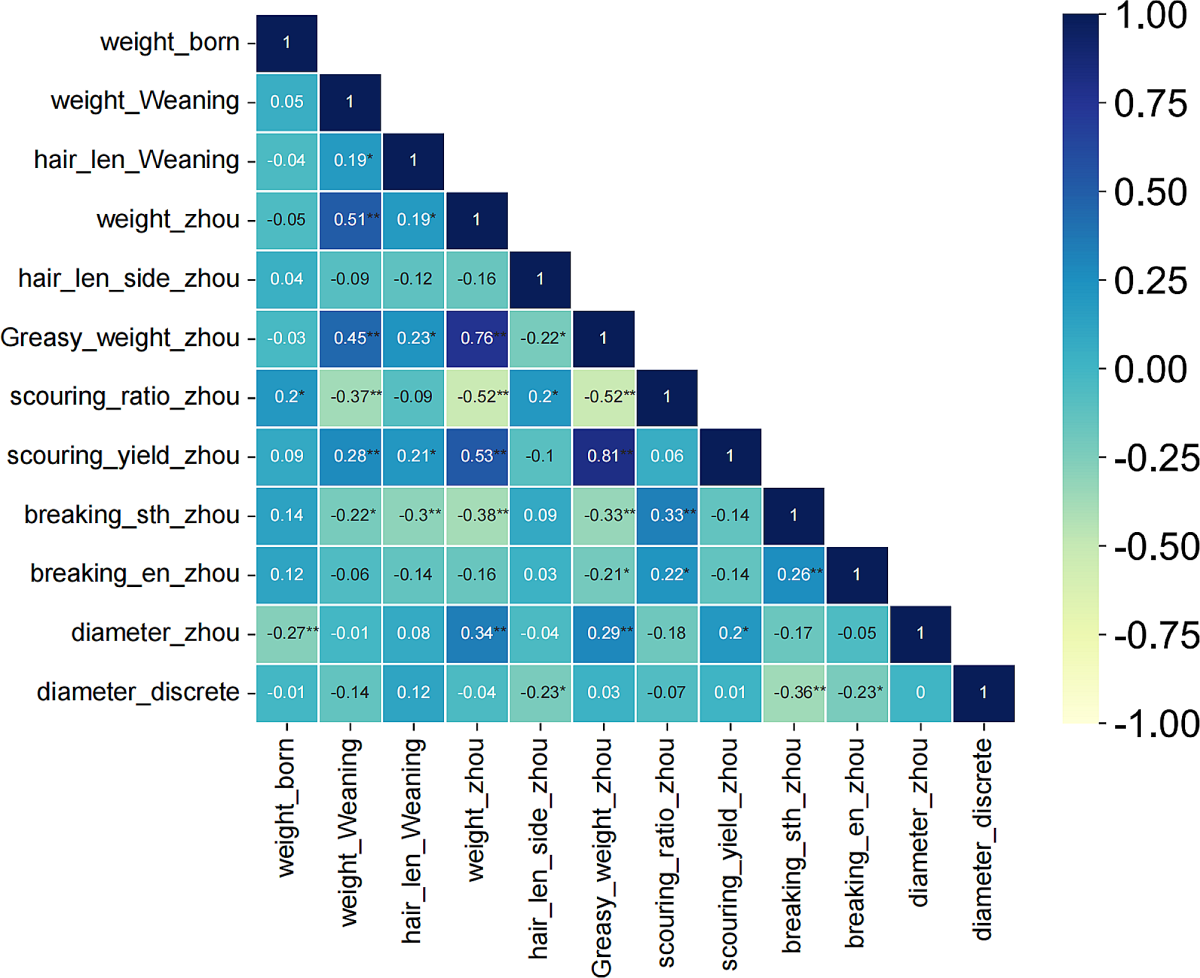
Heat map of Pearson’s correlation coefficient (R). Larger correlation coefficients are indicated using blue color and smaller correlation coefficients are indicated using yellow color. *,** indicate *p-value* < 0.05 and *p-value* < 0.01, respectively

The mapping family line in this study consists of 110 F_1_ generations, and the parameter names and means of the phenotypic traits related to body weight and wool production are shown in Table 1. The phenotypic traits of all individuals conformed to a normal distribution (Figure S1), characterized by continuous variation. The investigated traits can be specifically classified into three categories, i.e., traits related to body weight (including Newborn weight, Weaning weight, Yearling weight), traits related to wool production (including Yearling greasy weight, Yearling scouring ratio, Yearling scouring yield), and traits related to wool fiber characteristics (including Weaning hair length, Yearling side hair length, Yearling bundle fiber breaking strength, Yearling bundle fiber breaking elongation, Yearling wool fiber diameter, Yearling diameter discrete coefficient). From Fig. 1, Yearling greasy weight had the highest positive correlation with Yearling scouring yield (*r* = 0.81) and a high positive correlation with Yearling weight (*r* = 0.76); Yearling scouring ratio showed the highest negative correlation with both traits, Yearling greasy weight and Yearling weight (*r*=-0.52). Yearling greasy weight was significantly correlated with all traits (except Newborn weight and Yearling diameter discrete coefficient). Yearling weight, Yearling scouring ratio, Yearling bundle fiber breaking strength, and Weaning weight were significantly correlated with 7, 7, 7, and 6 traits, respectively. The correlations of Weaning weight and Yearling weight with traits related to hair production were all highly significant. Thus, subsequent QTL analyses should focus on loci associated with Yearling greasy weight, Weaning weight, and Yearling weight. Yearling wool fiber diameter and Yearling diameter discrete coefficient had low correlation with fewer traits. Thus, it can indicate to some extent that the wool diameter trait in Alpine Merino sheep is relatively independent and stable and less influenced by other traits.

**Table 1:** Phenotypic data for the F1 generation of Chinese Merino sheep

Term	Phenotypic trait	Parameter name	Mean±SD
Newborn	weight	weight_born	4.35±0.68 kg
Weaning	weight	weight_Weaning	28.32±3.19 kg
	hair length	hair_len_Weaning	4.35±0.57 cm
Yearling	weight	weight_zhou	47.50±10.53 kg
	side hair length	hair_len_side_zhou	10.67±0.87 cm
	greasy weight	Greasy_weight_zhou	4.67±1.03 kg
	scouring ratio	scouring_ratio_zhou	57.34±6.63%
	scouring yield	scouring_yield_zhou	2.64±0.46 kg
	bundle fiber breaking strength	breaking_sth_zhou	32.11±8.20 N/ktex
	bundle fiber breaking elongation	breaking_en_zhou	18.23±5.70%
	wool fiber diameter	diameter_zhou	18.40±1.55 μm
	diameter discrete coefficient	diameter_discrete	17.85±2.35%

### Genome resequencing

The DNA library was derived from 7 parents and 110 F_1_ generations, and 2,505 billion raw base (bp) (including 2,505.396 Gb raw data) was obtained by sequencing on the Illumina HiSeq™ PE150 platform. After quality control, a total of 16.3 billion Clean reads (including 2,449.341 Gb data, with a GC% of 44.17) were obtained, including 3.0 billion Clean reads from 7 parents and 13.3 billion Clean reads from 110 F_1_ generations. Per parent, an average of 428,376,791 Clean reads were obtained with a GC% of 44.29, all higher than 60Gb sequencing; per F_1_ generation, an average of 121,184,542 Clean reads were obtained with a GC% of 44.16, all higher than 15Gb sequencing (Table S1). The reference genome size was 2,587,507,373 bp, and the mapping rate of the seven parents to the reference genome ranged from 98.92 to 99.01%, with an average mapping rate of 98.97% (Table S2). The average coverage depth to the reference genome (excluding the N region) was 19.22X (Table S2). The mapping rate of 110 F_1_ generations to the reference genome ranged from 98.94 to 99.34%, with an average mapping rate of 99.18% (Table S2). The average coverage depth of the reference genome (excluding the N region) was 6.78 X (Table S2). The results were expected and can be used for subsequent mutation detection and related analysis.

### SNP discovery

**Fig. 2:**
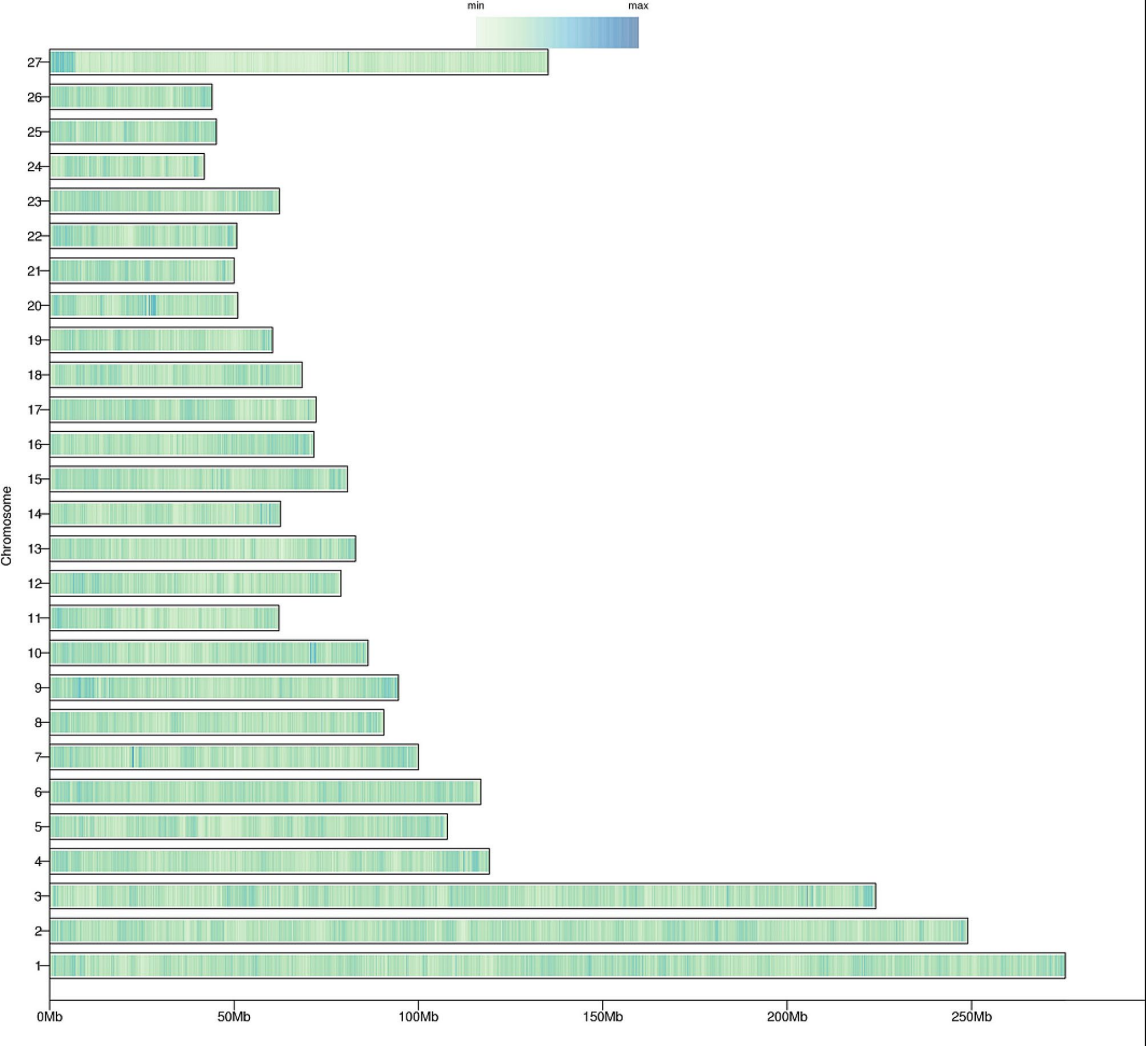
SNP density profiles. Each bar represents a chromosome, and the color depth of each bar represents the number density at the location, the darker the color

The SNP density map (Fig. 2) visualized the distribution of SNPs on chromosomes. We can find that each chromosome is colored. Thus, we can conclude that each chromosome was detected completely, and no large segments were missed. Meanwhile, the SNP density map demonstrated the variation in the genome. Dark blue regions indicated that there may be more variants, while light green regions may be relatively conserved. The statistics of the ANNOVAR annotation results are shown in Table 2. The statistical analysis of SNPs provided a variety of different types of SNPs and their distribution in the genome. As can be seen from Table 2, the variants mainly occurred in the intergenic region with 20,662,257 SNPs. Secondly, the variants occurred in the intronic region (11,796,384 SNPs) and the exonic region (216,209 SNPs); the exonic variants comprised 122,182 synonymous SNPs and 92,684 nonsynonymous SNPs. This SNP dataset will be valuable for promoting biological research and breeding of Alpine Merino sheep.

**Table 2:** Statistics of the SNP test results

	Category	Number of SNPs
	Upstream	185,714
Exonic	Stop gain	1192
Exonic	Stop loss	151
Exonic	Synonymous	122,182
Exonic	Non-synonymous	92,684
	Intronic	11,796,384
	Splicing	1127
	Downstream	183,366
	upstream/downstream	4487
	Intergenic	20,662,257
	ts	23,716,804
	tv	9,767,889
	ts/tv	2.428
	Total	33,484,693

### SNP marker genotyping

We selected loci with the same SNP and identical genotypes in seven parents as parental markers (without distinguishing between heterozygous and pure loci) and obtained a total of 33,282,477 markers. We inferred the maternal genotype using these parental markers and the F_1_ generation QTL analysis method (Table 3). Based on this table, we judged that there were no more than two genotypes in the offspring when the paternal markers were pure. Therefore, when the offspring have one or two genotypes, we label the locus nnxnp. When the father is labeled heterozygous, there are two possible scenarios for the offspring: (1) if the mother is pure, the offspring will have two genotypes; therefore, when the offspring have one or two genotypes, we label them lmxll; and (2) if the mother is heterozygous, the offspring will have three genotypes; therefore, when the offspring genotypes are one, two, or three, we label them hkxhk. The SNP typing results are shown in Table S4. The genotyped progeny markers were screened, and after biased segregation analysis, 14,942 valid markers were finally retained, which will be used for further linkage analysis.

**Table 3:** Genotypic composition and marker type statistics of parents and progeny

The F1-generation marker type	Father marker type	Mother marker type	The progeny marker type	Marker number
nnxnp	nn	np	nn, np	684
hkxhk	hk	hk	hh, hk, kk	11,268
lmxll	lm	ll	lm, ll	2990
Total				14,942

### Genetic linkage map

**Fig. 3:**
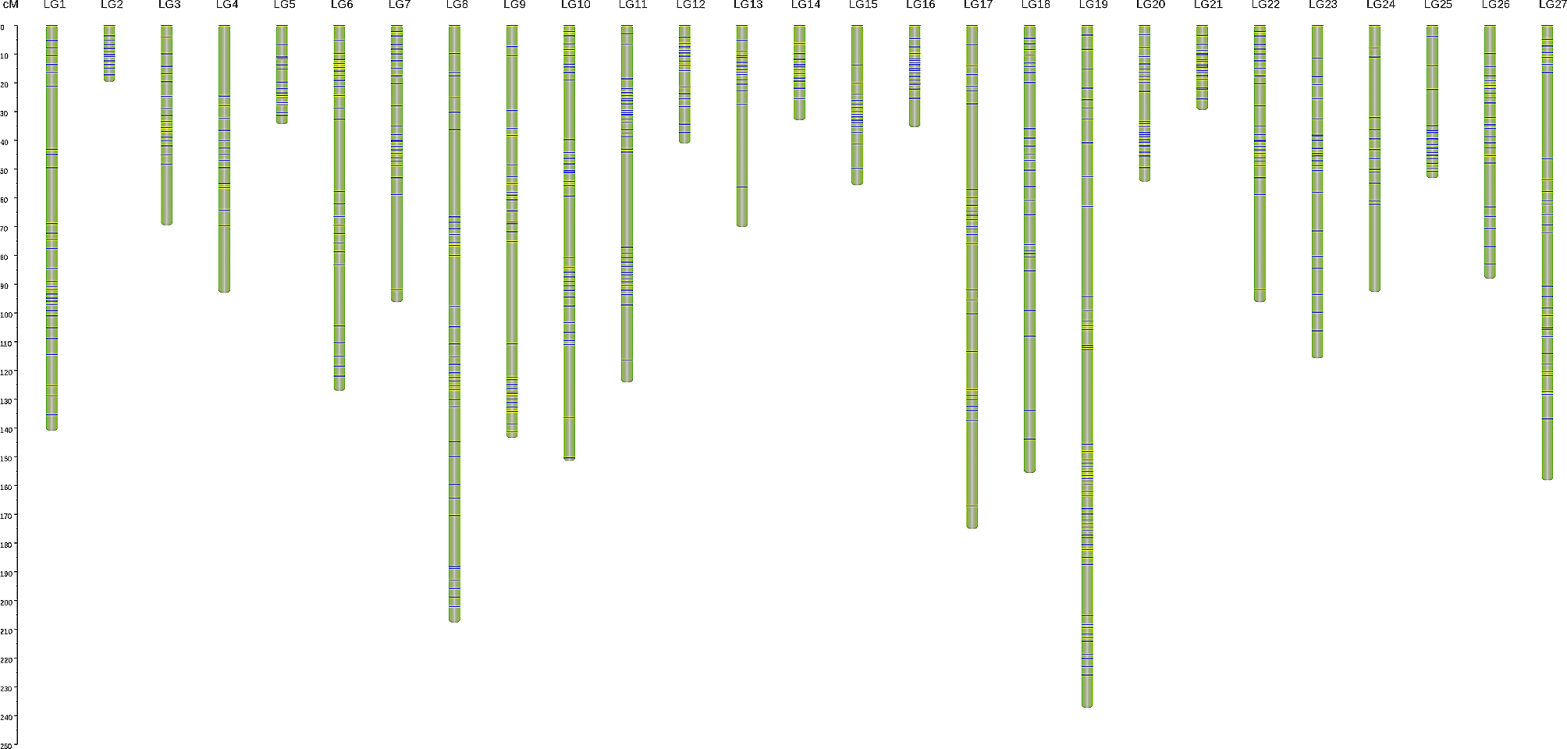
The Genetic linkage map (no marker number)

We first removed the markers that were seriously unable to be linked (i.e., we removed some markers with a linkage distance of 0). Then constructed a high-density genetic map by genetic linkage analysis. The number of SNP markers on each LG and the total length of genetic distance in each LG are shown in Table 4. Among them, 1871 SNP markers were localized to 27 LGs. The total genetic distance of this map was 2697.86 cM, with an average genetic distance per marker of 1.44 cM. The genetic distances of the LGs ranged from 19.62 cM (LG2) to 237.19 cM (LG19), with an average of 99.92 cM. On average, there were 69 markers per LG, with LG14 possessing the lowest marker density of 39 markers and LG1 the largest with 150 markers. Marker intervals ranged from 0 to 35.62 cM, with an average of 1.44 cM. The average genetic interval for markers in LG2 was the smallest at 0.24 cM, while the average genetic interval for markers in LG17 was the largest at 3.57 cM. The distribution of gaps (genetic distances between the markers) on each chromosome was counted, and the results are shown in Table 4. The Genetic linkage map (no marker number) is shown in Fig. 3 and Table S3. The Genetic linkage mapping (with marker number) is shown in Figure S2. The number of gaps with inter-marker genetic distance less than 5 cM was 1741 (Table 5), the number of gaps with inter-marker genetic distance between 5 cM and 10 cM was 75, the number of gaps with inter-marker genetic distance between 10 cM and 20 cM was 31, and the number of gaps with inter-marker genetic distance of larger than 20 cM was 24. The gaps between markers with a genetic distance of less than 5 cM accounted for 93.05% of the total number of markers, indicating that the markers were evenly distributed in the LGs.

**Table 4:** Statistics of genetic linkage groups

Group	marker_number	length	average_length	max_gap
lg1	150	140.93	0.94	21.98
lg2	83	19.62	0.24	3.63
lg3	77	69.51	0.9	21.27
lg4	69	93.02	1.35	24.61
lg5	52	34.34	0.66	6.76
lg6	73	127.05	1.74	25.21
lg7	51	96.26	1.89	33.1
lg8	82	207.57	2.53	30.4
lg9	149	143.41	0.96	35.62
lg10	53	151.4	2.86	25.51
lg11	67	124.14	1.85	33.11
lg12	45	41.06	0.91	6.07
lg13	88	70.11	0.8	28.74
lg14	39	32.98	0.85	7.47
lg15	89	55.62	0.62	13.82
lg16	69	35.38	0.51	10
lg17	49	175.02	3.57	29.79
lg18	53	155.61	2.94	25.83
lg19	84	237.19	2.82	32.8
lg20	74	54.33	0.73	10.42
lg21	75	29.38	0.39	3.78
lg22	51	96.26	1.89	33.1
lg23	51	115.79	2.27	13.43
lg24	43	92.66	2.15	30.47
lg25	43	53.12	1.24	12.51
lg26	58	88	1.52	15.27
lg27	54	158.11	2.93	30.08
total	1871	2697.86	1.44	35.62

Note: Group: Linkage group number; SNP markers: number of SNP markers; Map length: genetic distance length; Average distance (cM): average genetic distance; Max.gap (cM): maximum genetic distance between markers

**Table 5:** Statistics of the distribution of gap size across the chromosome in the genetic map

Group	<5 cM	5to10	10to20	>20 cM	ratio
lg1	142	5	2	1	94.67
lg2	83	0	0	0	100
lg3	74	2	0	1	96.1
lg4	64	3	0	2	92.75
lg5	51	1	0	0	98.08
lg6	68	3	0	2	93.15
lg7	47	3	0	1	92.16
lg8	68	10	3	1	82.93
lg9	143	2	3	1	95.97
lg10	48	1	1	3	90.57
lg11	63	1	2	1	94.03
lg12	43	2	0	0	95.56
lg13	85	1	1	1	96.59
lg14	37	2	0	0	94.87
lg15	85	3	1	0	95.51
lg16	68	1	0	0	98.55
lg17	41	3	3	2	83.67
lg18	44	4	4	1	83.02
lg19	74	4	4	2	88.1
lg20	73	0	1	0	98.65
lg21	75	0	0	0	100
lg22	47	3	0	1	92.16
lg23	40	9	2	0	78.43
lg24	39	2	0	2	90.7
lg25	40	1	2	0	93.02
lg26	52	5	1	0	89.66
lg27	47	4	1	2	87.04
total	1741	75	31	24	93.05

Note: Chr: the name of the chromosome;

<5 cM: the number of markers less than 5 cM;

5 to 10 cM: the number of genetic distance gaps between markers, between 5 cM and 10 cM;

10 to 20 cM: the number of genetic gaps between markers between 10 cM and 20 cM;

> 20 cM: number of gaps with genetic distance between markers greater than 20 cM;

Ratio: percentage of gap with genetic distance less than 5 cM to total number of markers;

### QTL localization of traits related to body weight and wool production

**Fig. 4:**
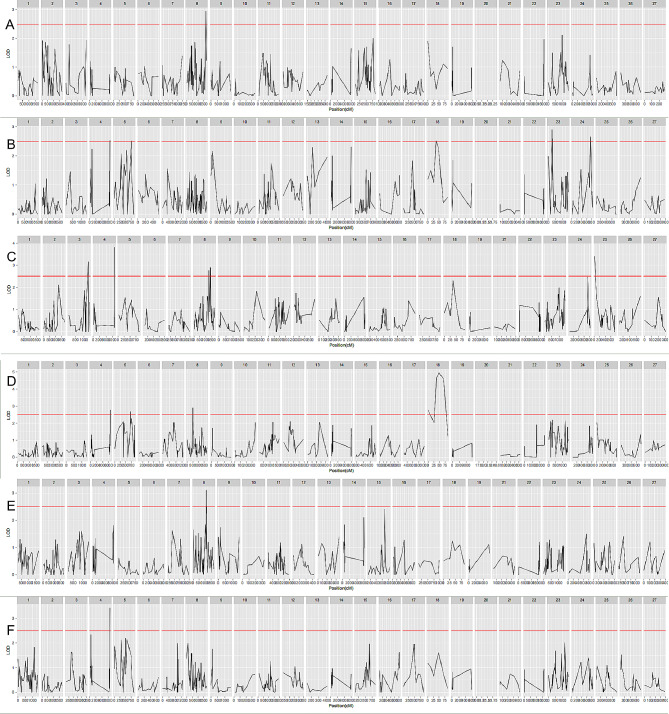
Results of phenotypic trait localization for body weight, wool production. A: Newborn weight; B: Weaning weight; C: Yearling weight; D: Yearling greasy weight; E: Yearling scouring yield; F: Yearling scouring ratio

**Fig. 5:**
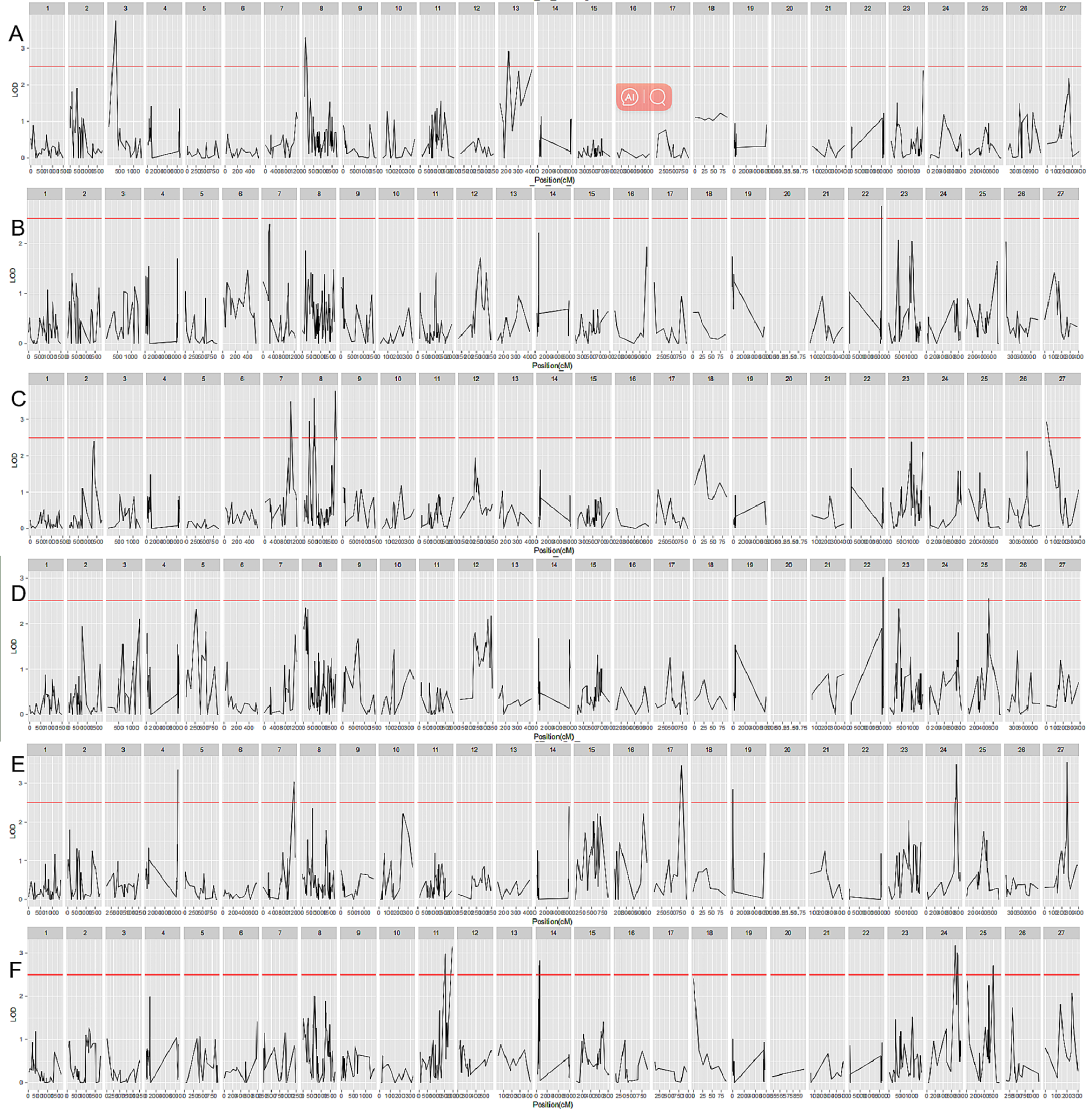
Results of phenotypic trait localization for wool fiber characteristics. A: Weaning hair length; B: Yearling side hair length; C: Yearling wool fiber diameter; D: Yearling diameter discrete coefficient; E: Yearling bundle fiber breaking strength; F: Yearling bundle fiber breaking elongation

QTL localization was performed by MapQTL 6.0 software for 12 traits, which were distributed over 17 LGs. Results of phenotypic trait localization for body weight, wool production, wool fiber characteristics are showed in Figs. 4 and 5. The LOD values of these QTLs ranged from 2.51 to 4.96. Among them, the highest LOD for Yearling greasy weight was 4.96. Among the three weight-related traits, we identified a total of 12 QTLs, of which one was associated with Newborn weight, six with Weaning weight, and five with Yearling weight. For the three traits related to wool production, a total of eight significant QTLs were identified, including five QTLs related to Yearling greasy weight, two QTLs related to Yearling scouring yield, and one QTL related to Yearling scouring ratio. For the six traits related to wool fiber characteristics, a total of 22 QTLs were localized, namely, 2 Yearling diameter discrete coefficient-related QTLs, 6 Yearling wool fiber diameter-related QTLs, 1 Yearling side hair length-related QTL, 3 Weaning hair length-related QTLs, 7 Yearling bundle fiber breaking strength-related QTLs, and 4 Yearling bundle fiber breaking elongation-related QTLs. The largest number of QTLs were distributed on LG8, 11 in total, encompassing six different phenotypic traits covering three categories: body weight, wool production, and wool fiber characteristics. It should be noted that QTL intervals with overlapping traits were found on LG4, including Yearling greasy weight, Weaning weight, Yearling weight, Yearling scouring ratio, and Yearling bundle fiber breaking strength (Table 6). In addition, on LG8, overlapping QTL intervals with Yearling wool fiber diameter and Newborn weight were also found (Table 6).

**Table 6:** QTLs detected related to body weight and wool production traits

Pheno	Group	Position	LOD	Pheno	Group	Position	LOD
diameter_discrete	22	16135.89	3.01	weight_Weaning	24	763.746	2.66
diameter_discrete	25	505.996	2.55	weight_zhou	3	1256.262	3.16
diameter_zhou	7	990.84	3.49	weight_zhou	4	6623.697	3.81
diameter_zhou	8	1705.566	3.77	weight_zhou	8	1280.701	2.77
diameter_zhou	8	296.075	2.96	weight_zhou	8	1403.228	2.9
diameter_zhou	8	578.657	3.58	weight_zhou	25	17.002	3.4
diameter_zhou	8	594.559	2.83	scouring_yield_zhou	8	1103.696	3.1
diameter_zhou	27	0	2.94	scouring_yield_zhou	8	1122.386	2.51
Greasy_weight_zhou	4	6623.697	2.77	scouring_ratio_zhou	4	6623.697	3.43
Greasy_weight_zhou	5	707.084	2.69	breaking_sth_zhou	17	760.836	3.45
Greasy_weight_zhou	8	493.75	2.89	breaking_sth_zhou	19	145.807	2.83
Greasy_weight_zhou	18	0	2.76	breaking_sth_zhou	20	51.435	2.66
Greasy_weight_zhou	18	47.844	4.96	breaking_sth_zhou	24	741.399	3.49
hair_len_side_zhou	22	16197.91	2.74	breaking_sth_zhou	27	262.046	3.53
hair_len_Weaning	3	335.839	3.75	breaking_sth_zhou	4	6623.697	3.35
hair_len_Weaning	8	35.863	3.3	breaking_sth_zhou	7	1193.374	3.03
hair_len_Weaning	13	218.317	2.93	breaking_en_zhou	11	1570.878	2.98
weight_born	8	1705.566	2.94	breaking_en_zhou	11	1956.564	3.14
weight_Weaning	4	6623.697	2.53	breaking_en_zhou	14	488.524	2.71
weight_Weaning	5	762.906	2.52	breaking_en_zhou	14	618.899	2.82
weight_Weaning	18	36.687	2.52	breaking_en_zhou	24	701.012	3.17
weight_Weaning	23	266.112	2.9	breaking_en_zhou	24	751.614	3
weight_Weaning	23	289.093	2.59	breaking_en_zhou	25	591.848	2.71

Note: Pheno: trait name; Group: linkage group number; Position (cM): genetic location of the LOD peak in the qtl section; LOD peak in the LOD: qtl section;

## Discussion

SNP-based high-density genetic linkage mapping can not only improve the QTL localization effect of phenotypic traits [47–50], but also obtain molecular markers that are closely linked to the associated QTL [50, 51]. Thus, a deeper understanding of the relationship between genes and phenotypes can be obtained [52], providing theoretical guidance for molecular MAS in Alpine Merino sheep.

### High-density genetic linkage map

The populations used for QTL localization are generally categorized into permanent populations (including recombinant inbred lines (RIL) [53], haploid (HAP), and double haploid (DH)) and temporary populations (subgeneration (F_1_), subgeneration (F_2_), and backcross (BC)) [54]. Currently, F_1_ generation families are used to construct genetic linkage maps for most aquatic species, *Hemibagrus wyckioides* [55], *Larimichthys crocea* [56, 57], *Nibea albiflora* [47], *Aristichthys nobilis* [58]. The population used in this study is the F1 generation, and since the parents have both pure and heterozygous genotypes, it is thus possible to obtain rich genetic information and construct a genetic linkage map in a relatively short period of time.

The total length of the genetic linkage map of the F1 generation of Alpine Merino sheep constructed from 1,871 SNPs was 2,697.86 cM, the average length of each LG was 99.92 cM, and the average interval of the markers was 1.44 cM. The uniform distribution of markers indicates that the map constructed in this study has high marker density and high accuracy. The atlas contained 27 LGs, consistent with Alpine Merino sheep’s chromosome number. This indicates a high degree of confidence in this map. It is the latest high-density, high-resolution genetic linkage map among all Merino sheep breeds in recent years, providing an essential tool for further fine localization of molecular markers.

### QTL for traits related to body weight and wool production

Several studies have been conducted to identify practical QTL for many milk production [59–61], growth rate and weight [24], and disease resistance [62, 63] traits in sheep utilizing linkage maps. Among them, there are relatively more QTL studies on milk and disease resistance, while similar studies on body weight are fewer and QTL studies on wool yield and wool characterization are few. Therefore, this study fills in the lack of QTL information on body weight and wool yield and trait traits in existing studies and provides new information and insights for genetic improvement and optimal selection in sheep.

In this study, we completed the QTL for weight and wool production-related traits in Alpine Merino sheep based on the high-density genetic linkage map constructed by WGR. Firstly, among the three weight phenotypic traits, we detected a total of 12 QTL associated with Newborn weight, Weaning weight, and Yearling weight in LG3, LG4, LG5, LG8, LG18, LG23, LG24, LG25, in total. This suggests that genes from different chromosomes may affect the same trait. This result is consistent with previous studies that weight-related traits are influenced by multiple QTL and multiple genes [24, 64–66]. Meanwhile, among the QTL associated with body weight, three are located in LG8, two in LG4, and two in LG23, which is relatively dispersed. This implies that multiple genetic linkage groups influence Alpine Merino sheep’s body weight. Of all the QTL associated with body weight, this study and the Awassi-Merino [24] backcross progeny study found overlap on chromosomes 3, 23, and 24. However, in the comparison at one year of age (approximately 52 weeks), only chromosome 3 showed overlap in both studies. Whereas, in the comparison with wild bighorn sheep [67], chromosomes 23 and 24 were found to overlap with weight-related QTL in this study. In comparison with Scottish Blackface lambs [68], on the other hand, only chromosome 24 was shown to have overlapping QTL. QTL overlap on chromosome 24 was present among different breeds, suggesting that chromosome 24 may carry key genetic factors that are essential for weight regulation. Meanwhile, a comparison with the Merino sheep half-sibling test showed that the QTL localized to the birth weight of the two sheep on different chromosomes, respectively chromosomes 8 and 3 [69]. Secondly, for the three traits related to wool production, we identified eight important QTLs for Yearling greasy weight, Yearling scouring ratio, and Yearling scouring yield at LG4, LG5, LG8, and LG18. While Merino sheep half-sibling test [69] was different from the present study, the QTL for the three traits were distributed on chromosomes 1,3,11. In this study, three of these QTLs were located on LG8, suggesting that a major effector gene in LG8 may affect wool production. In addition, both the Yearling scouring yield QTL and the Yearling greasy weight QTL were located at LG8, but the QTLs did not overlap, and there was a significant Pearson correlation between the two. This suggests that a joint regulatory network may affect Yearling scouring yield and Yearling greasy weight, which may include multiple genes within the same linkage group. Even if these genes are not in the same location, their effects may be interrelated through complex regulatory pathways, leading to correlations between the two traits. Finally, a total of 22 QTLs were localized to LG3, LG4, LG7, LG8, LG11, LG13, LG14, LG17, LG19, LG20, LG22, LG24, LG25, LG27 out of the six traits related to wool fiber characteristics; these included Yearling diameter discrete coefficient, Yearling wool fiber diameter, Yearling side hair length, Weaning hair length, Yearling bundle fiber breaking strength, and Yearling bundle fiber breaking elongation. Among them, most of the six QTLs associated with Yearling wool fiber diameter were located in LG8, suggesting that there may be principal effector genes affecting wool fiber diameter in some regions of LG8. In this study, QTL for bundle fiber breaking strength was present on chromosome 4, which was consistent with the QTL results of the Merino sheep half-sibling test [69]. And QTLs for diameter discrete coefficient and bundle fiber breaking elongation were found on chromosome 25, which was the same as the results of the synthetic breed INRA401 study [70]. In addition, we found that the wool of male sheep may be slightly finer than that of female sheep [71–74], and the QTL associated with wool diameter was also present on the interlocking cluster of sex chromosomes (LG27). Also, sex hormone-related genes such as androgens (e.g., testosterone) are present on the sex chromosomes (e.g., AR is located on the X chromosome [75]). Thus, in combination with some previous studies [9, 75–77], it can be hypothesized that sex differences in wool fiber diameter may be due to differences in hormone levels between the two sexes as well as genes located on the sex chromosomes. In addition, we found that the Yearling diameter discrete coefficient and the Yearling wool fiber diameter did not share a common LG, which could lead to a less stable wool diameter in the selective breeding of fine-wool sheep, with possible consequences for the uniformity and quality of the product. Although the QTL localization for the Yearling wool fiber diameter and Yearling greasy weight traits had a common LG, there was a lack of overlapping regions, which could lead to difficulties in co-breeding for the wool diameter and greasy weight traits. Localization of the Weaning hair length and Yearling bundle fiber breaking strength traits was sporadic, with no more than 1 QTL region on the same LG, suggesting that the traits may be controlled by multiple genes on multiple chromosomes.

Five overlapping regions of QTL were found to be simultaneously associated with weaning weight, Yearling scouring ratio, Yearling bundle fiber breaking strength, Yearling greasy weight, and Yearling weight. In addition, two overlapping regions of the QTL were concurrently associated with Newborn weight and Yearling wool fiber diameter. These results suggested that the overlapping QTL may control multiple traits and that the genes in these regions had significant pleiotropic effects [78]. This pleiotropic effect facilitates co-breeding for multiple traits. Also, the presence of overlapping regions may be due to significant phenotypic correlations between phenotypic traits (Fig. 1). Based on Pearson’s correlation coefficient and its significance analysis, most of the correlations between these five traits (Yearling greasy weight, Yearling scouring yield, Yearling scouring ratio, bundle fiber breaking strength, bundle fiber breaking elongation) were consistent with the Merino sheep half-sib trial [69]. There was no correlation between the traits of body weight and wool in the Merino sheep half-sib trial. However, this study additionally found that Yearling wool fiber diameter showed significant correlations with Newborn weight, Yearling weight, Yearling greasy weight, and Yearling scouring yield. Also body weight trait showed significant correlation with eight traits of wool yield and wool characteristics. Significant correlations between different traits in the overlapping regions of these QTL revealed genetic linkages between the traits. This has important implications for breeding and genetic improvement as multiple traits with the same genetic basis can be precisely selected and improved to enhance overall performance.

There are some shortcomings in this study. First, the phenotypic data were not comprehensive enough to include other important traits such as body size, wool fiber curvature, and second greasy weight, which may affect the accurate localization and interpretation of QTL. Second, the sample size was not yet sufficiently adequate and needs to be further increased to improve the credibility and statistical validity of the study. Meanwhile, the small number of parents may not provide genotypic information of sufficient diversity, which may affect the accuracy and reliability of QTL localization. Finally, it is impossible to use breeding values for the study because we did not count non-genetic factors [79].

## Conclusions

Alpine Merino sheep genetic linkage mapping is constructed through a denser set of markers that locate QTLs that will be more accurately associated with body weight, wool production traits, filling in the lack of information on QTLs for body weight, wool production and trait traits. Breeders will be able to more accurately select good parents carrying favorable QTLs and cross them to pass on beneficial gene combinations more efficiently to the next generation, resulting in better progeny. This will accelerate the improvement process of ultra-fine and meat-type Alpine Merino, improving productivity and animal quality. This method of genetic improvement is of great significance for the development of animal husbandry and the improvement of herd performance.

### Methods

#### Resource family and phenotypic data

At the Huangcheng Farm, a cooperative breeding unit of the Lanzhou Institute of Husbandry and Pharmaceutical of the Chinese Academy of Agricultural Sciences, we selected seven healthy, breed-typical parents and 110 F_1_ generation individuals from new breeds of Alpine Merino sheep. All samples were provided with high-quality feed and sufficient clean water; a suitable and comfortable feeding environment, including appropriate space, dry housing, and appropriate temperature range; regular health checks and vaccination programs; hygiene maintenance, reasonable exercise, and rest; breeding management; and regular equipment inspection and maintenance. Phenotypic data for weight and wool production traits, such as newborn, weaning, and yearling identification index, were also measured for individual samples. Also, we performed normality tests on the phenotypic data provided. The phenotypic data was statistically analyzed using SPSS 22.0 [80] software. We used the Pearson correlation coefficient to measure the degree of linear correlation between two continuous variables. For correlations between multiple features, we constructed a correlation matrix. We displayed the magnitude of the Pearson correlation coefficient using a color-coded heatmap to visualize the strength of the correlation between different features. Heatmap was plotted by https://www.bioinformatics.com.cn (last accessed on 10 Nov 2023), an online platform for data analysis and visualization [81]. Finally, blood samples were collected from 117 Alpine Merino sheep and stored at -20 ℃ temperature for DNA extraction.

### Library construction and sequencing

To construct the DNA library, the DNA samples were randomly interrupted into fragments of 350 bp length by a Covaris crusher (S220, Covaris, USA). The TruSeq Library Construction Kit (Illumina, San Diego, CA, USA) was used to construct the library. DNA fragments undergo end repair, addition of a polyA tail, attachment of sequencing adapters, purification, PCR amplification, and other steps to complete the entire library preparation process. The constructed library was sequenced by Illumina HiSeq™ PE150 (Illumina, San Diego, CA, USA) to produce raw reads. The sequencing data were strictly filtered to obtain high-quality clean reads to ensure the quality of information analysis. The raw data were filtered as follows: (1) Removing reads with ≥ 10% unidentified nucleotides (N); (2) Removing reads with > 50% bases having phred quality < 5;(3) Removing reads with > 10 nt aligned to the adapter, allowing ≤ 10% mismatches; (4) Removing putative PCR duplicates generated by PCR amplification in the library construction process (read 1 and read 2 of two paired-end reads that were completely identical). Sequencing data were compared to the reference genome (Oar_v4.0 (GCA_000298735.2)). PE reads from parental and progeny Clean data were compared to the reference genome using BWA (v 0.7.17) [82] comparison software (settings: mem -t 4 -k 32 -M -R). The results were formatted into SAM/BAM files using SAMTOOLS [83] (settings: -bS -t). In addition, potential PCR duplications were removed using SAMTOOLS command “rmdup”. Perl scripts were used to count comparison rates and coverage. The results were ranked using SAMTOOLS (settings: sort) for variant detection.

### SNP detection

SNP mainly refers to DNA sequence polymorphisms caused by variations in a single nucleotide at the genomic level, including transitions and inversions of individual bases. We used SAMTOOLS for population SNP detection. In order to reduce the error rate of SNP detection, the following criteria were selected for filtering: (1) the number of reads supported by SNPs was not less than 4; (2) the mass value (MQ) of SNPs was not less than 20. The results of the SNP site detection in the samples were annotated by using ANNOVAR [84] software.

### Genotyping and SNP identification

There are seven known fathers and no mothers, so we chose as parental markers (without distinguishing between heterozygous and heterozygous loci) loci that are genotypically identical at the same SNP locus in all seven fathers. We used the obtained parental markers and the F_1_ generation QTL analysis method to infer the maternal genotypes. We then categorized the parental markers. After typing, the offspring markers were screened for the following criteria: (1) Abnormal base checking. For abnormal bases that appear in the progeny but do not exist in the parent, we regard them as deletions. (2) Completeness filtering. Markers with genotypes covering at least 84% of all individuals in the progeny are filtered, and we will filter markers with poor genotypic integrity coverage. (3) Segregation distortion [85] filtering. Candidate markers were filtered for segregation distortion using the chi-square test, with a threshold *p* of 0.01 set for segregation distortion.

### Linkage group construction

For the high-quality genetic markers obtained after screening, Joinmap 4.1 [86] software was used for genetic map construction. (1) Linkage groups were divided, and the LOD values were set from 2 to 50. It is known that the genome of sheep has 27 pairs of chromosomes, so 27 linkage groups with more marker numbers were retained based on the number of markers in each linkage group as the basic linkage groups for subsequent analysis; (2) Maximum Likelihood algorithm was used to sort the markers in each linkage group; (3) Kosambi function was used to calculate the genetic distances between markers.

### QTL localization of traits related to body weight and wool production

In this study, we used MapQTL 6.0 [87] software to locate QTL for body weight and wool production traits. The analysis process consisted of first using the PT (Permutation Test) algorithm to determine the LOD threshold for each phenotype. We did not include covariates or cofactors in the analysis. Then, QTL location was performed using the Interval Mapping algorithm in the MapQTL software. Finally, based on the threshold value obtained from the PT, the QTL regions larger than the threshold value in the QTL location results were extracted as the significant QTL regions corresponding to the trait.

### Electronic supplementary material

Below is the link to the electronic supplementary material.

Supplementary Material 1

Supplementary Material 2

Supplementary Material 3

Supplementary Material 4

Supplementary Material 5

Supplementary Material 6

## Data Availability

The datasets generated and analyzed in the current project (PRJNA1083948) are deposited in the NCBI SRA repository (http://www.ncbi.nlm.nih.gov/bioproject/1083948).

## Abbreviations

GWAS: Genome-Wide Association Study
QTL: Quantitative Trait Loci
MAS: Marker-Assisted Selection
LG: Linkage Group
SNP: Single Nucleotide Polymorphism
WGR: Whole Genome Resequencing
NGS: Next Generation Sequencing
