# Supplementary figures and images for: Construction of a high-density genetic map and QTL localization of body weight and wool production related traits in Alpine Merino sheep based on WGR
Source: BMC Genomics. 2024 Jun 27;25:641. doi: 10.1186/s12864-024-10535-4 (PMC11212225; doi:10.1186/s12864-024-10535-4)

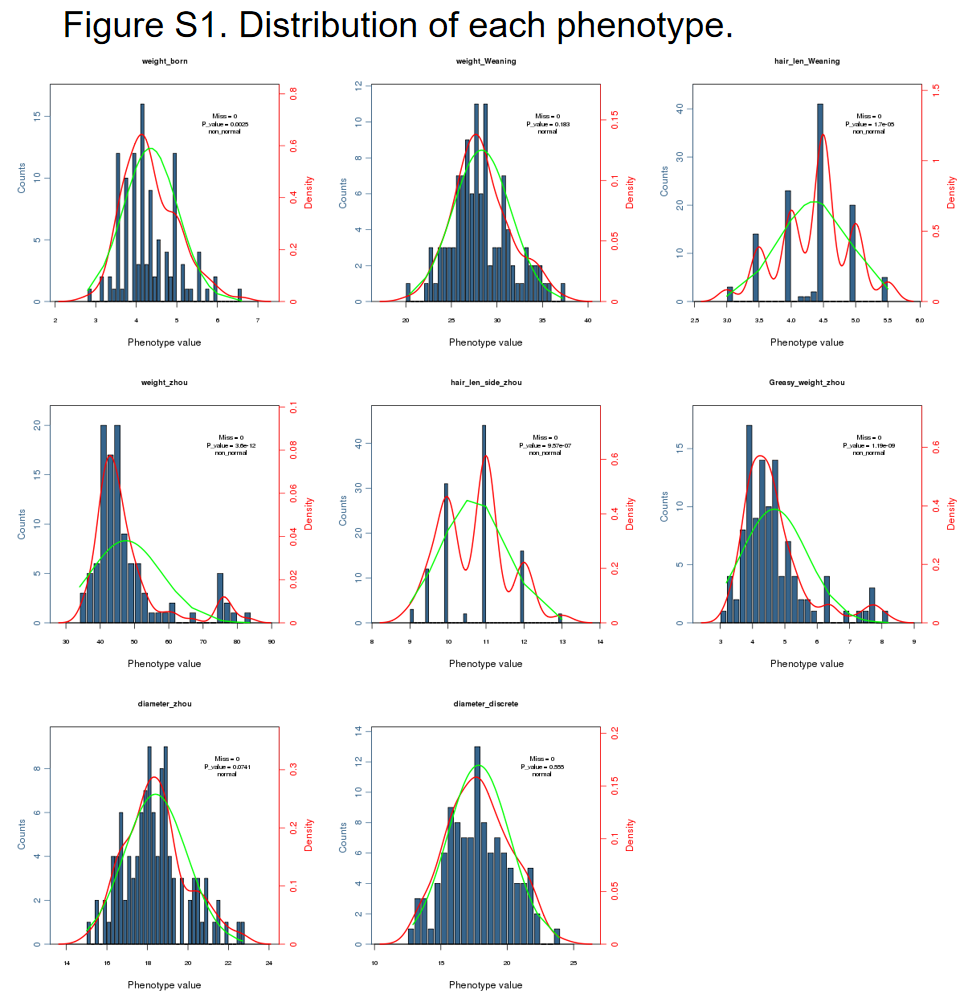

Supplement: Supplementary file 1 — Supplementary Material 1 [file 12864_2024_10535_MOESM1_ESM.png]

Figure S2. The Genetic linkage map (with marker number).

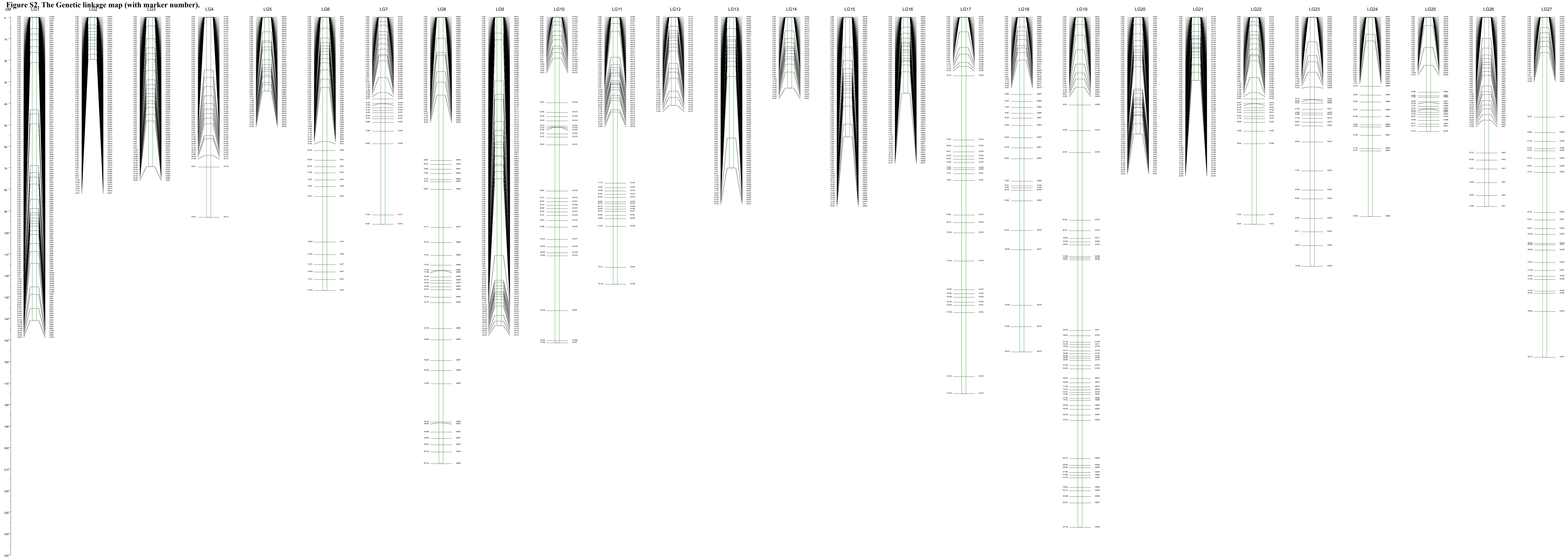

Supplement: Supplementary file 4 — Supplementary Material 4 [file 12864_2024_10535_MOESM4_ESM.pdf]
